# Items

**Published:** 1907-05

**Authors:** 


### ITEMS.

The United States Civil Service Commission announces an examination on June 13-14, 1907, to secure eligibles from which to make certification to fill at least five vacancies in the position of medical interne (male), at $600 per annum each, with maintenance, in the Government Hospital for the Insane, Washington, D. C., and vacancies as they may occur in any branch of the service requiring similar qualifications. For information in detail address the United States Civil Service Commission, Washington, D. C.

---

Messrs. Battle & Co., Saint Louis, have issued recently No. 1 of the series of eighteen illustrations of dislocations, which will be a complement to the series of long bone fractures. This pamphlet contains a chart showing bilateral dislocation of jaw, and will be sent free to physicians on application.

Occupation Dusts.—The American Institute of Social Service (*Mobile Med. & Surg. Jour.*) has received from Dr. Sommerfield, a physician and scientist of Berlin, a valuable anti-tuberculosis exhibit for the department of industrial hygiene in its Museum of Security. There are 45 vials containing as many different kinds of dust, mineral, animal and vegetable, produced in our various industries. The same number of photographs show how these various dusts appear under the microscope. Extremely realistic models in wax, colored to life, represent human lungs as they are affected by occupational dusts, other models show normal lungs for comparison; while still others show the effects of industrial poisons on the system.

At the meeting of the Executive Committee of the American Institute of Social Service held in April at the Players' Club as the guests of Mr. Richard Watson Gilder, announcement was made that the Scientific American, through a desire to co-operate with the work of the Institute in promoting an American Museum of Safety Devices, would give annually a gold medal to be awarded by the Institute for the best device for preventing accidents.

An advisory committee of the editors of the great technical papers was organised to co-operate with the Institute in the work of protecting life and limb. As at present constituted the advisory committee consists of fourteen representatives from the Scientific American, Iron Age, American Machinist, Railway and Locomotive Engineering, Automobile, Electrical World, Street Railway Journal, Dry Goods Economist, Electrical Age, Railway Gazette, and Engineering and Mining Journal.

---

Legislation for Vital Statistics.—Cressy L. Wilbur, Washington, D. C., declares (*Texas Medical News*) that the condition of vital statistics in the United States is a reproach to the country. He states that the importance of vital statistics, both from a legal standpoint and for the purpose of effective sanitary administration, is altogether overlooked by us. He calls attention to the states in which registration has been enforced, and shows in tabular form the states which constituted the registration area in 1900 and those which have been added since. He refers to the work of the American Medical Association in this matter and quotes from the transactions of the association for 1848 to show that the accurate registration of vital statistics was one of the first objects which the association desired to promote. He takes up in detail the necessary provisions of a registration law for deaths and calls attention to the evils of non-enforcement of the law when one exists. The county system of attempting to collect vital statistics he terms pernicious. It has never yet, he declares, successfully collected them in a single state of the many in which it has been tried, and he considers it one of the stumbling blocks that are continually thwarting efforts to secure adequate legislation. He mentions the work of the Bureau of the Census and in a footnote enumerates some of its publications relating to this subject which are available.—*Journal A. M. A.*

